# Gender relations and women's reproductive health in South Sudan

**DOI:** 10.3402/gha.v9.33047

**Published:** 2016-11-28

**Authors:** Sumit Kane, Matilda Rial, Anthony Matere, Marjolein Dieleman, Jacqueline E.W. Broerse, Maryse Kok

**Affiliations:** 1 KIT Health, Royal Tropical Institute, Amsterdam, The Netherlands; 2 Independent Consultant, Wau, Western Bahr el Ghazal, South Sudan; 3 School of Public and Environmental Health, University of Bahr el Ghazal, Wau, Western Bahr el Ghazal, South Sudan; 4 Athena Institute for Research on Innovation and Communication in Health and Life Sciences, Faculty of Earth and Life Sciences, VU University Amsterdam, Amsterdam, The Netherlands

**Keywords:** sexual and reproductive health, gender, South Sudan, reproductive health, women’s health, reproductive decisions

## Abstract

**Background:**

In South Sudan, women disproportionately bear the burden of morbidity and mortality related to sexual and reproductive health, with a maternal mortality ratio of 789 deaths per 100,000 live births.

**Design:**

A qualitative study was conducted to analyze how gendered social relations among the Fertit people affect women's ability to exercise control over their reproductive lives and thereby their sexual and reproductive health. Transcripts of 5 focus group discussions and 44 semi-structured interviews conducted with purposefully selected community members and health personnel were analyzed using Connell's relational theory of gender.

**Results:**

Women across all age groups report that they have little choice but to meet the childbearing demands of husbands and their families. Women, both young and old, and also elders, are frustrated about how men and society are letting them down and how they are left to bear the reproductive burden. The poverty and chronic insecurity in South Sudan mean that many men have few sources of pride and achievement; conformity and complicity with the hegemonic practices accord both security and a sense of belonging and privilege to men, often at the expense of women's reproductive health.

**Conclusions:**

Inequalities in the domestic, social, and economic spheres intersect to create social situations wherein Fertit women's agency in the reproductive realm is constrained. In South Sudan, as long as economic and social opportunities for women remain restricted, and as long as insecurity and uncertainty remain, many women will have little choice but to resort to having many children to safeguard their fragile present and future. Unless structural measures are taken to address these inequalities, there is a risk of both a widening of existing health inequalities and the emergence of new inequalities.

## Introduction

Globally, women bear a disproportionate burden of morbidity and mortality related to sexual and reproductive health (SRH). De Francisco et al. (1) argued that individuals’ SRH is shaped by the nature of intimate and family relations set within kinship structures, community institutions, and relations. They added that how women experience their sexual and reproductive situation and health is embedded within a variety of gendered social relations – relations with their intimate partners, immediate family, community, and, ultimately, broader society. Gender and health researchers have argued for studies to investigate the complex interactions between gender and other structures of social inequalities to understand health situations (2, 3). Others have pointed out that the complex nature of these interactions seems to hinder the scholarship on this subject and have urged researchers to take on this challenge (1, 4); this article attempts to do this in the context of the SRH of women in South Sudan.

South Sudan became an independent country in July 2011. The long war preceding independence has destroyed much of the public systems such as education, health care, and infrastructure. The continued sporadic episodes of violence and chronic insecurity in many parts of the country have also disrupted the social fabric. According to the Human Development Report of 2015, South Sudan is ranked 169 out of 188 countries and territories for human development (5). Economically, according to the World Bank, although South Sudan has much potential, there is great inequality and widespread poverty, with 51% of the population living below the poverty line (6). Almost 72% of the population is under the age of 30 years; most of them are unemployed, unskilled, rural, and female (6). Illiteracy is widespread: Around 84% of all women are illiterate. Over half (57%) of all households in South Sudan are female headed (7). The health system is weak, with severe shortages of health workers and poorly functioning health facilities (8, 9). South Sudan has a maternal mortality ratio of 789 deaths per 100,000 live births (10), a contraceptive prevalence rate of just 4.7%, and a teenage pregnancy rate of 34.5% (11, 12). These indicators highlight the gravity of the SRH situation in South Sudan; although reliable data disaggregated by state and ethnic group are not available, it is reasonable to assume that the SRH situation is similar throughout the country and across all ethnic groups. South Sudan is home to more than 50 ethnic groups. Although at the national level the Dinka and the Nuer people constitute the biggest ethnic groups, in some states, other ethnic groups tend to predominate. For instance, in the state of Western Bahr el Ghazal (WBeG), the main ethnic group is the Fertit, a moniker used to refer to a loose conglomeration of more than 23 non-Dinka, non-Arab, non-Fur, and non-Luo people (13) who freely intermarry. Unlike the Dinka and the Nuer people, who are pastoralists, the Fertit people are predominantly agriculturists involved in subsistence farming.

This article presents an analysis of how gendered social relations among the Fertit people of the WBeG state of South Sudan interact and intersect to affect Fertit women's ability to exercise control over their reproductive choices and decisions and thereby their SRH. Such an analysis which exposes how gendered social relations and practices in the domestic, local social, and economic spheres contribute to shape Fertit women's sexual and reproductive agency and health can inform the development of locally appropriate public policy and public health responses. This insight can also be potentially useful for other parts of South Sudan with a similar social context.

## Methodology

Data are drawn from a 2-year study exploring SRH decision-making and actions, conducted within the context of a larger SRH project implemented in South Sudan from 2013 to 2016. The SRH project aims to improve reproductive health outcomes in three states by supporting the state ministries of health and their development partners to implement the National Sexual and Reproductive Health Strategic Plan 2013–2016. Project activities are geared toward achieving three complementary objectives: improving the availability, accessibility, and use of quality SRH services; strengthening capacities at all levels of the ministries to deliver quality SRH services; and generating knowledge for locally appropriate and effective approaches to improve SRH. As part of the latter, a qualitative study was conducted in Wau county to gain insight into various factors shaping people's SRH choices, decisions, and actions. Data were collected through focus group discussions (FGDs) and semi-structured interviews (SSIs) with a variety of purposefully selected informants, as detailed in Table 1.

**Table 1:** Overview of the study participants and data collection

Method	Profiles of the study participants	Number of activities (number of participants)
FGD	Community members: Female 18–35 years (not in union^a^)	1 (8)
	Community members: Female 18–35 years (in union)	1 (8)
	Community members: Male >35 years	1 (8)
	Community members: Male 18–35 years	1 (8)
	Health workers	1 (6)
SSI	Community member: Female 18–35 years (not in union)	5
	Community member: Female 18–35 years (in union)	6
	Community member: Male 18–35 years	6
	Community member: Female >35 years	6
	Community member: Male >35 years	4
SSI with key informants	Traditional birth attendants	4
	Traditional leaders	3
	Health facility personnel	5
	State SRH managers	2
	NGO representatives	3

a Participants were either in union or not in union at the time of the study; we articulate relationship status this way because in WBeG, one would publicly state his or her status as married only if the relationship was formalized either in a traditional ceremony or in the church. For the sake of convenience, we use the terms ‘married’ and ‘unmarried’ in the article.

FGD, focus group discussion; NGO, non-governmental organization; SRH, sexual and reproductive health; SSI, semi-structured interview.

Topic guides for FGDs and SSIs were developed using de Francisco et al.'s (1) framework of ‘Circles of Influence Affecting Sexual and Reproductive Decisions’. According to the framework, SRH of men and women is shaped by overlapping spheres of influence within the family, community, and broader society. SRH of individuals is affected by the nature of intimate and family relations, including gender relations set within kinship structures, community institutions, and other social relations, which are, in turn, nested in broader social institutions, power structures, and ideologies. According to the framework, within these overlapping spheres of influence, individuals and social groups occupy positions of relative advantage or disadvantage with respect to their access to information and other resources; this shapes their capacity to make decisions and has important implications for their own and others’ SRH. The framework allowed the topic guides to cover a wide range of issues affecting choices, decisions, and actions related to SRH at household, community, and the broader societal relations level. The topic guides for health and other workers included questions along the same lines, but with a view to exploring their perspectives on the situation. The FGD and SSI topic guides for the community members were prepared in English and translated into Wau Arabic (by investigators MR and AM). The topic guides were defined further through consultations with stakeholders, and after pre-testing in the study sites, and were also adapted iteratively as the study progressed. The FGDs and SSIs with community members were conducted in Wau Arabic, and interviews with health and other workers were conducted in English.

Connell's (14–16) relational theory of gender is used to analyze and explain how gendered social relations shape Fertit women's agency in the reproductive realm and thereby their SRH. The theory of gender relations understands gender as simultaneously involving ‘economic relations, power relations, affective relations and symbolic relations …’ (14); the enduring patterns of these social relations being what social theory calls ‘structures’ (p. 73). Connell (15) argued that an analysis of how social relations shape a particular social phenomenon or social situation should do so through an examination of the interplay between these structures , that is, the ways they interact and shape each other and produce social situations. Connell's relational theory of gender was chosen because it steers clear of assumptions of social categories, hierarchies, and individual-centeredness; instead, by focusing on social relations and their social construction as antecedents of gendering, Connell's framework allows one to approach any social context openly. Further, Connell's framework does not assume social relations to be ahistorical; as Oyewumi (17, p. 1) recommended, it allows one to ‘ask questions about the meaning of gender and how to apprehend it in particular times and places’. Many African gender theorists acknowledge Connell's work as an exception to the often Western-centric and universalist theoretical perspectives on gender (18, 19).

## Study sites

The study was conducted in Wau county in the WBeG state of South Sudan. Two locations were selected based on homogeneity of the residents (all Fertit). The two locations represented two different settings: one, a rural area with a tight-knit community less exposed to non-Fertit cultural influences, and the other, the town of Wau with a community that has greater interaction with other communities and possibly less tightly knit. The *a priori* assumption behind choosing these two locations was that perhaps within the same social group (the Fertit), depending on the setting, gender relations and the enforceability of social norms might be different and that this might have different consequences for women's SRH.

## Sampling, recruitment of study participants, and data collection

Community members were purposefully selected with the help of village elders, community health workers from a local non-governmental organization (NGO), and the county health department; they were selected based on their potential to provide rich insight into the study subject. The study was explained first to the village elders, who then allowed us to talk to people in their community. This due process ensured unrestricted access to the community. The actual selection of study participants was done by the research team. Among the community members, only those aged 18 years and above were included in this study; a separate, but linked study has been conducted among adolescents. We purposefully categorized the participants into those aged 18–35 years and those over 35 years of age. The assumption was that the two age groups might experience gender relations and their SRH-related consequences differently.

Data collection began with FGDs among the community members, which was followed by SSIs to obtain more in-depth understanding. For FGDs with community members, the participants were homogenous in terms of ethnicity, age, and marital status, while diversity was sought in terms of social and economic status (based on inputs from elders related to social identity, ownership of assets such as bicycles and level of education). However, given the widespread poverty in WBeG, most study participants can be considered poor. Those involved in FGDs were not involved again in the SSIs. All FGDs and SSIs with the community members were conducted in the local language, Wau Arabic.

Key informants were also purposefully selected based on their active SRH-related role within the study community and the local health system; they were identified through the initial stakeholder consultations. As elaborated in Table 1, key informants included health facility personnel from the local health centers (many of whom were from the Fertit community), traditional leaders, traditional birth attendants, state- and county-level SRH service managers, and NGO representatives.

Data were collected from October 2014 to April 2015 by research team members who hailed from the study area, were fluent in the local language (Wau Arabic), and had experience with conducting qualitative research. FGDs and SSIs with some health workers, managers, and NGO representatives were carried out in English, as some of them hailed from other parts of the country and did not speak Wau Arabic (English is the official language of South Sudan). Data were collected until theoretical saturation was reached and no new insight emerged. This was possible to assess, as at the end of each day of data collection, the research team debriefed and discussed the emerging findings. SSIs and FGDs were digitally recorded, translated from Wau Arabic to English (where applicable), and transcribed verbatim. The translations were independently checked by one of the coauthors (MR) to ensure that the translations were accurate. In total, 5 FGDs (with 38 participants) and 44 SSIs were conducted.

## Data analysis

A thematic content analysis was conducted using Connell's relational theory of gender. Transcripts were analyzed using NVivo 10 software; this was performed in parallel by three researchers (SK, MK, and MR). Emerging themes were identified through a process of discussion and argumentation within the research team.

Analysis was refined through follow-up interviews with two study participants in each study site (*n*=4) – one Sultan (a traditional leader among the Fertit and in South Sudan at large) and one local resource person – and through a workshop involving community health workers, health facility personnel, and SRH service managers.

## Results

Four key themes emerged. The first theme articulates the high symbolic value attached to childbearing and paternity in Fertit society; the antecedents of the high symbolic value as discussed by study participants are presented. The second theme discusses the status of women in Fertit society, the power relations in the domestic and family spheres generally, and in light of the symbolic value attached to childbearing, and examines the effects of these relational arrangements on Fertit women's reproductive agency. The third theme builds on these two themes to highlight how broader societal and political circumstances have undermined the social compact among the Fertit and how this amplifies the unequal gender order, further undermining women's ability to exercise their reproductive agency and thereby their reproductive health. The fourth theme presents evidence of how social and gender relations are constantly in flux and are being actively constructed (14) by Fertit women in relation to others at the individual and the societal level. It shows how Fertit women are leveraging opportunities presented to them by the particular setting they are in at this time, to influence the gender order – although probably in a very limited way.

## Symbolic value attached to fertility, childbearing, and paternity

Among the Fertit, as the following quotes show, fertility and childbearing are seen as markers of respectability and responsibility. Having many children – expanding one's family and spreading the family name – is a key aspect of manhood.

A good man in the community is responsible. When he is a responsible person in the community, he is well respected and has his children. When a man does not have children, in the community he is seen as not responsible. (B – SSI Female <35 – Unmarried – 1)

If the boy becomes a man he needs to extend his family by getting married and having children. In this way the family expands and they become well known to the people. (A – SSI Male <35 – 3)

Further, among the Fertit, children are seen as a means of carrying on one's family name. Those men who do not father many children are seen as wasting their lives. Symbolically, children are also seen as a means of replacing lost (dead) family members; in fact, children are often designated as replacing specific lost relatives. In the last three decades, given the war and widespread human losses, this social practice has become a powerful force to entrench the symbolic value attached to childbearing; the result being the furtherance of the masculine hegemony, often at the expense of women's agency in the reproductive realm.

If a man stays without having children we think that he may die and leave no name behind. Whatever he owns will be a waste. (B – SSI Male <35 – 1)

Participant: ‘They think of their family members who died and they want to have children so that their family can expand and they become proud in society as a big family’. Interviewer: ‘So men want children to carry their names?’ Participant: ‘Yes, just for the name, but they cannot handle raising children’. (A – SSI Female <35 – Unmarried – 3)

Fertit women are expected to give birth to as many children as the man and his family members wish, because it allows inheritance and the continuation of the man's family name; this is an entrenched social norm. Consistent with the quote below, study participants across age, sex, marital status, and geography noted that a woman is meant to bear children for her husband's family and that women do not really have a say in this matter.

Our relatives see that birth allows inheritance and if you do not want to give birth, men do not agree. They (husband and his relatives) don't agree when women decide they do not want to have children. (A – SSI Female >35 – 3)

## Unequal status and power relations between the sexes

The Fertit, like the other South Sudanese ethnic groups, are patriarchal; men have the power to decide on all aspects of the family and in society at large, and women's position is subordinate to men (20–23). The Fertit consider the notion of being respectable, and worthy, of great importance; boys and men count, whereas women do not. This was a cross-cutting theme mentioned by all the participants; for instance, in an FGD with young women, there was consensus when one of the participants pointed this out; probing by the facilitator led to a discussion wherein it emerged, as the following quote illustrates, that this was indeed a given among the Fertit.

A boy is respected because he is a boy. But if you are a girl, you are just a girl. You are not respected. (B^1^ – FGD Female <35)

The following sections will illustrate how this structurally lower status accorded with women and girls in Fertit society intersects with and also shapes other structural forces, and prevents women from exercising their agency in many social realms, particularly in the reproductive realm. In Fertit society, the decision-making power on matters related to sex and reproduction rests with the man and his family.

The man is the man, and this is his responsibility … so the decision (on matters related to sex and reproduction) should come from the man. (A – SSI Traditional Leader – 2)

A key feature of Fertit society is that the family is a consanguinally based unit built around a core of brothers and sisters (blood relations). The wife is not seen as part of the family, but as an outsider whose role is to bear children for the man's family. The following interaction in an FGD among young women shows how, in Fertit society, the woman's *raison d’être* is her ability to produce children for the man and for his family.

Participant 1: ‘If you are married and already living with your husband and do not have a child, the husband can leave you and tell you to go back to your family’.

Participant 2: ‘His relatives will come and argue that why you are not getting pregnant and sometimes that you are barren. The man's relatives will complain why is this woman brought and eating our food for free if she is not going to deliver children’.

Participant 1: ‘The relatives will tell the husband to leave you and go and get another woman who can have children’.

Participant 3: ‘Or the (man's) relatives themselves will go and get a wife for their son’. (B – FGD Female <35)

Women who wish to differ and make their own reproductive choices are subject to serious social pressures, particularly from the husband's family. Women are often at risk of being abandoned by their husbands, and given that economic opportunities are so few in South Sudan, particularly for women, they are left with no choice but to submit to the demands of the husband and his family. As the following quotes from two young women highlight, these unequal power relations are reflected within intimate partnerships too. Many women see themselves as beholden to the man who brought them into his house. Not only do women need to consider it their duty to listen to their husband, but not refusing to have sex is also seen as essential to being able to keep one's husband and as key to preventing him from going to other women. This surrender of agency is thus driven by a confluence of the social position of women in society, the fear of the man bringing home sexually transmitted infections, and also, given the harsh economic situation of South Sudan, the fear of abandonment.

A woman's responsibility is to listen to her husband, do the house work and look after your children, and you don't roam randomly outside. You listen to the person who brought you at home. (A – SSI Female <35– Married –1)

The man insists on sex, and if the woman refuses he will go look for another woman. So if you want to keep your husband, you need to have sex with him whenever he wants (…) So women try to please their husbands; that is why they become pregnant without planning. (B – SSI Female <35– Unmarried – 1)

In WBeG, and across South Sudan, women outnumber men, are poorer than men, and are less educated than men. Economic opportunities are few for women, and many women are dependent on men (6, 12). Our findings show that many women are left with no choice but to resort to competing with each other to keep their men by using their capacity to bear children for him and his family. The following interaction in an FGD among young married women highlights the insecurities they experience; it shows how social structural forces intersect to compromise their agency in the reproductive realm.

Participant 1: ‘The woman will say it is enough and the man will say he wants more and there will be a lot of pulling between them here’.

Participant 2: ‘If you don't want …. You go to your home’. Interviewer: ‘What happens after she returns to her family? …. The husband will get another wife?’

Participant 1: ‘The husband will get another wife … Again after you’.

Participant 2: ‘It won't take long … he will bring another woman’. (B – FGD Female <35)

The following example, given by one of the health care providers, is a particularly incisive reflection of the complicated situation many Fertit women are in:

Women say that they have to give birth so that their husbands don't leave them. You find men having two wives, and each wife thinks that childbirth will make the man not go away. Once a woman came here and her husband is in Khartoum and she has 10 children and she has a tubal ligation [a non-reversible method to prevent pregnancy involving surgery on fallopian tubes]. I think she has fibroids [fibroids are benign masses in the uterus], but she told me that she feels she is pregnant and she wants to give birth because she has a new husband and she needs to give birth for him. (A – SSI Health Facility Personnel – 2)

## Breakdown of traditional social contracts as amplifiers of gender inequalities

There is some evidence that civil wars and chronic conflicts may increase the risk of death and disability through the breakdown of social norms and practices which maintain social order, and that women and children bear most of the long-term burden of the consequences of this breakdown (24, 25). Our findings also reveal that the long war and ongoing insecurity have undermined the traditional checks and balances in Fertit society. In the traditional Fertit family structure, the children belong to the man and his family, and the man and his family are expected to take responsibility for the wife and her children. Mechanisms, both formal and traditional, for maintaining and enforcing this social contract, wherein men take responsibility for their family, have been weakened, to the detriment of Fertit women's agency in general, and in the reproductive realm in particular.

A recurring theme throughout the study, among both the old and the young, was of men increasingly abrogating their responsibilities toward their wives and children. As the following quote from an FGD involving older women illustrates, in the current context, Fertit society is falling short by sanctioning traditionally unacceptable behaviors – causing much hardship to women.

Giving birth during our time is not like giving birth now. During our time when a woman gives birth you work on the farm … your husband also supports you, he brings everything. But childbirth nowadays … the boys never care about their wives, when she is pregnant whether she is eating or not or whatever she is doing is not his problem … This brings a lot of hardship to women. (A – FGD Female >35 – Married)

This loss of social control was poignantly articulated by an elderly NGO worker who hailed from the Fertit community; she said the following:

It is true our time was not like this. Because when we were young girls of course you will love a boy, but that boy is afraid to touch you. You will write letters … I love you … you will sew handkerchiefs for your boyfriend, but he will never touch you … because he is afraid. (SSI NGO Worker)

They [boys] go get girls pregnant and … there is nothing girls can do … they drop out of school and suffer raising their children, even [having to] go and sell goods in the market. (A -- SSI Female <35– Unmarried – 3)

As the above quote from a young, unmarried woman shows, some women feel helpless; they feel that they have been left alone to bear the burden of pregnancy, childbirth, and childrearing; they feel let down by society. Health workers themselves, Fertit women, born, raised, and living in the area for a long time, also recognized this breakdown of social control in their society. They acknowledged that there was widespread frustration among women about men's behaviors and about society's failure to exercise social control. They highlighted various instances of men's abandonment of women, adding that this was a major social problem in WBeG and also beyond.

I will tell you the truth, between our time and now there is a big difference. During our time when a boy reaches puberty and is still living with his parents he cannot go on his own and choose a girl it has to be through your family, your father or your mother. But now young boys who have not yet become responsible, date girls, rape them and impregnate them and later on refuse to have them as their wives. (B – SSI Health Facility Personnel – 2)

In Fertit society, the children belong to the man's family and are his responsibility; the woman is an outsider and does not belong to the family. A Fertit woman's position in the family structure is in many ways contingent on the effectiveness of social control and on men acting responsibly. During fieldwork, women across age groups pointed out that not only the traditional marriage practices were gone but also the social expectations and responsibilities that accompanied the traditional way of organizing a family were no longer enforceable in the current context, to women's great disadvantage.

At this time there is no marriage you just cohabit and if you don't want him any more then there is nothing; you just leave. If he removes your belongings it's ok … if not you take them yourself and leave. This is what is happening here … not like before. (A – SSI Female <35– Unmarried – 2)

Oyewumi (26), citing Sudarkasa (27), has argued that gender relations in African societies should be examined within the frame of a ‘consanguinally-based family system built around a core of brothers and sisters-blood relations’, wherein the spouses are considered outsiders and therefore not part of the family, instead of the Western frame of the conjugally based family built around a couple. She argues that doing so allows one to understand the status of the woman in relation not merely to her spouse but to his family, which consists of him and his siblings. Consistent with Oyewumi's point, our findings show that Fertit women see this abrogation of responsibilities as not only their husband's failure but also as a failure of his family to keep their part of the bargain and as a failure of the society to enforce it.

## Influencing the gender order

A key theme we recognized throughout the study was that this abrogation of men's responsibility and the failure of Fertit society to check it are catalyzing a situation wherein women are taking responsibility and decisions into their own hands. One might argue, as the following quotes from younger female participants indicate, that they are in the process proudly pushing back and challenging the unequal gender order in Fertit society – albeit in a small way.

Women do anything, any kind of business that will bring income so that children can have food … women will do it. For example in my house I bake bread and brew alcohol and I eat with my children if our supply finishes I go and buy more and we can have something on the table. I save my money to support my children in school. This time I registered my children in schools. … It is not their father. (A – SSI Female <35– Unmarried – 2)

‘The men nowadays will make you bear a lot of children and they don't take responsibility. That is why women say go find another wife to give birth to you, me … I am done with childbirth’. (A – SSI Female <35– Unmarried – 3)

All three Sultans interviewed also recognized this social situation. They were clearly disappointed with many men's conduct, particularly about men not taking their social responsibilities seriously. As the following quote by a Sultan shows, they also rued how many women were increasingly taking their lives into their own hands.

Like I have just mentioned men fail to take responsibility (…) In our community in South Sudan I now see that women are more responsible than men because when you wake up in the morning you see women going to work, to farms, to different places … and women … they are doing better in raising children than men. These days people are failing in life and the failure comes from men. Because when you give birth to five children you need to send them to school, treat them, and do so many things, so every morning men have to go and find ways of feeding children (and they don't) …. (A – SSI Traditional Leader – 2)

Based on what we were told by some participants, and based on the work of South Sudanese social scientists (21, 23), we expected the Sultans to be the conventional guardians of Fertit patriarchy, devoted to justifying the status quo. On the contrary, as the above quote also illustrates, they seemed to not just acknowledge these societal failures, but, based on our insight from the fieldwork, were also quite open to a situation where women run the show. The Sultan's views were echoed by many male study participants; these views, perhaps, signal the existence of much more fluid and flexible social relations in Fertit society than we were able to uncover in this work.

## Discussion and conclusions

A key feature of Fertit society is the high symbolic value it attaches to fertility and childbearing. The family is a consanguinally based unit built around a core of brothers and sisters (blood relations), with the wife not seen as part of the family, but as an outsider whose role is to bear children for the man's family. Another key feature, which is linked to the structure of the Fertit family, is the notion that the children belong to the man and his family and that the children's upkeep is their responsibility. This responsibility extends to the children's mother, but is tacit, and in some ways contingent on her ability to continue to bear children. These social structures serve as the context within which gendered relations are enacted in Fertit society to create social situations wherein Fertit women's agency in the reproductive realm is constrained. We discuss women's accounts of constrained agency within three broad perspectives on fertility choices and decisions around fertility in contexts like that of South Sudan, to arrive at conclusions. We reflect on these accounts by locating them within broader discussions on the privileges of motherhood and childbearing in the sociopolitical context of South Sudan, by examining women's accounts of men's conduct within a broader understanding of men's imperatives to conform to the hegemonic masculine order, and, finally, by discussing how these explanations are possibly shaped by the overarching structural forces related to chronic insecurity and emerging changes in the economic order. In doing so, we also arrive at conclusions of relevance to SRH policy and practice in the WBeG state of South Sudan.

Green and Biddlecom (28) warned researchers, particularly those examining decision-making in the reproductive realm, against a simplistic problematization of men's role in women's reproductive health; they argued that ‘men as well as women may have financial motives for sex, because children may legitimate partners’ claims to one another's resources’. Although this was not made explicit by study participants, we did find evidence of women pandering to the paternity claims of multiple men simultaneously, to ensure their own and their children's financial and social protection. Without in any way condoning the unequal and unfair treatment of women, Green and Biddlecom's thesis allows one to see many Fertit men's actions as being socially constructed and a consequence of, and a means toward sustaining, actively or complicitly, the accrual of what Connell calls the ‘patriarchal dividend’ (29). The dividend refers to a series of material practices, which include political, cultural, economic, and social advantages over women and men who do not confirm to the dominant form of masculinity. In the context of South Sudan, given the chronic insecurity, opportunities for economic development and social achievement for men are few, and so are the resources to confront entrenched hegemonies. Therefore, in an environment where many men have few sources of pride and achievement, conformity and complicity with the hegemonic practices accord both security and a sense of belonging and privilege – clearly a safe strategy for many men, given the current context of South Sudan.

Caldwell and Caldwell (30) urged those studying fertility and related decision-making in sub-Saharan African societies to recognize that, unlike in Western and Oriental societies, in sub-Saharan Africa, the overarching emphasis in society remains on ancestry, descent, and family lineage. The experience and understanding about the cost of fertility are fundamentally different; they contended ‘that high fertility does not carry economic penalties, while the foreigner's experience has been very different’. Furthermore, historically, they argued that the demographic reaction in the period following chronic conflict and insecurity involving large-scale social disruption and human loss, is a rise in fertility (31); this thesis has been well established over the last two decades (32, 33). This is likely to be so for South Sudan as well.

Caldwell (30, 34) further argued that in contexts with insecurity, poverty, and uncertainty, women, by having many children, are in fact in some ways exercising their agency, however constrained it might be. There is a large body of literature that contends that children are a valuable resource for poor people, particularly poor women for whom children are a source of prestige, offer an old-age insurance policy in face of insecurity, and also serve as extra hands to do much of the labor-intensive domestic and subsistence work (35). Palmer (36), building on Caldwell's thesis, concluded that in contexts of insecurity and uncertainty, where there are few possibilities to plan for a better future, ‘women may retreat into their traditional role of motherhood for securing labour assistance and old age support’. Contrary to this thesis, we found that the social norms among the Fertit are such that the children are normatively seen as assets of the man and his family, and women do not generally have the social space to claim them as a resource, nor as an old-age insurance policy in the face of insecurity; in a separate article, we dwell in detail on social norms among the Fertit (37). We contend that Fertit women's narratives should be understood as being shaped by the social structures they inhabit. Fertit women are socialized to not expect and publicly stake a claim to their children as being theirs and as being their resources; instead, their statements actually reproduce the social structure by reaffirming that the children they bear are for the man and his family. This perhaps explains why, in our data, women never explicitly claimed their children as their resource and are conspicuously silent about it. This explanation mirrors Oyewumi's (26) assertion that, in many African societies, the family is not built around the conjugal couple but is consanguinally based and built around a core of brothers and sisters (blood relations), with the spouses considered outsiders.

During the interviews, the study participants did not refer much to insecurity and violent conflict. This could be a reflection of their acceptance of the situation as the normal state of affairs and taking it for granted, or it might reflect the relative security that WBeG has enjoyed. That said, evidence from conflict studies shows that chronic insecurity and conflict undermine women's agency in all social realms, including the reproductive realm (33); that it affects women's health disproportionately (24, 25); and that it does so through further entrenchment of hegemonic patriarchy. The absence of social order and rule of law in WBeG and, in South Sudan at large, the result of chronic insecurity and conflict are key drivers of exaggeration of the inequalities in social and economic relations in Fertit society. It allows those in positions of relative power, usually men, to act with impunity at the expense of liberties, opportunities, and dignity of the weak, often the women. Caldwell and Caldwell, in their seminal work in the 1980s and the 1990s, observed and predicted that the new economic and social order would undermine this high-fertility system (30). Their contention was that in the new economic order, a move away from a subsistence agriculture-based economic model to a more mixed economic model would increasingly expose the incompatibility between high fertility and the high costs of raising children and the ever-greater costs of education. This demographic trend is being observed in some sub-Saharan African societies, but not in others (38, 39); what will happen in South Sudan remains to be seen. In a separate article, we have discussed how social norms shape reproduction and family planning-related decisions among the Fertit (37); we have specifically examined how norms are evolving, and why.

In conclusion, Fertit women's reproductive choices and decisions are not totally their own, nor are they only the function of their capability to make decisions, but, in the current context and time, are shaped as much by the society in which they live. This has important implications for public health policy and practice. It is important to recognize that the Fertit people desire to have children and that such a desire is not only rooted in the Fertit culture; this is in line with the demographic experience in other such contexts. Any policy which does not take this into account is not only likely to fail but also will not serve people's needs and might backfire. On the one hand, the consanguinally based family structure among the Fertit is unique and has its own social logic and relationship dynamics. However on the other hand, this social logic is failing Fertit women, and they are feeling let down by society. Interventions for improving Fertit women's SRH should necessarily address this problem. To have an impact on all these fronts, interventions not only need to target women but also need to engage actively with the complex social situation by including and targeting men, their families, and Fertit society at large.

The return of peace and the re-establishment of law and order in South Sudan will improve access to health care services and will most likely help improve Fertit women's reproductive health. But the emergence of a non-agrarian economic model, and the expected oil-driven economic upturn, is likely to be a mixed bag for women, particularly poor women. An adult female illiteracy rate of 84%, coupled with decades of constrained agency due to war and structurally unequal gender social and economic relations, means that women in WBeG are less likely than men to be able to benefit from the opportunities that will emerge with the return of peace. Therefore, in WBeG, and in South Sudan generally, as long as economic and social opportunities for women remain constrained, and as long as insecurity and uncertainty remain, many women will have little choice but to resort to having many children to safeguard their fragile present and future. The high fertility and poor health care services will together continue to imperil the health and lives of women in South Sudan. Unless structural policy measures are taken to address these inequalities, the disadvantaging of women, particularly poor women, may be further amplified, and there is a risk of both a widening of existing health inequalities and the emergence of new inequalities. These conclusions are consistent with the conclusions of a recent review of gender equity and SRH in Eastern and Southern Africa (40).

## Ethics and consent processes

The study was approved by the Independent Ethics Committees of KIT Royal Tropical Institute, Amsterdam, and of the national Ministry of Health of the Government of South Sudan. Administrative approval was given by the WBeG state Ministry of Health. Informed written consent was sought from all the participants; for those who could not read, the consent form was read out to them, and their consent was recorded. Confidentiality was maintained throughout, and steps were taken to anonymize the data and to minimize risk of accidental disclosure or access by unauthorized third parties. Because the broader study included questions about the local SRH services and the responsiveness of providers, steps were taken to ensure that identities of the participants were not revealed to the local health workers. At the beginning of the consent process, the participants were informed of their right to refuse to answer questions they might find intrusive. Furthermore, given the sensitive nature of the topic, there is a risk of opening up hitherto closed, yet painful chapters and experiences in the person's life. To ensure support if such a situation arose, a trained counselor was available, as were medical referral services. No such situation requiring counseling or medical referral emerged during the data collection.

## Footnotes

1 Prefix ‘A’ refers to the participants from study site around Wau town; ‘B’ refers to the participants from the rural location in Wau county. During data collection, we realized that the distinction was immaterial, as the town is merely a larger village.

## Paper context

Decades of war, ongoing insecurity, and structurally unequal, gendered social and economic relations, and the consanguinally based family unit context are key intersecting drivers of poor sexual and reproductive health of Fertit women in Western Bahr el Ghazal, South Sudan. Meaningful and sustainable change can occur only if health policy and program interventions explicitly address these drivers, target not only women but also equally and actively engage men and their families.
